# Mind and Muscle: A Retrospective Study on the Management of Psychiatric Comorbidities in Patients With Fibromyalgia Treated With Pregabalin Versus Milnacipran

**DOI:** 10.7759/cureus.103154

**Published:** 2026-02-07

**Authors:** Angelica Arshoun, Andrew S Murdock, Eduardo D Espiridion

**Affiliations:** 1 Psychiatry, Drexel University College of Medicine, Reading, USA; 2 Psychiatry, Drexel University College of Medicine, Philadelphia, USA

**Keywords:** chronic pain management, fibromyalgia, milnacipran, opioid medication, pregabalin

## Abstract

Background: Fibromyalgia syndrome is a debilitating chronic disease with symptoms of pain and fatigue that profoundly impact patients’ quality of life. This disability can lead to the development of psychiatric comorbidities, causing a compounding decline in overall well-being. Pregabalin and milnacipran are options for patients seeking symptomatic relief, but we hypothesized that psychiatric outcomes differ between patients with fibromyalgia treated with milnacipran versus pregabalin.

Methods: TriNetX, a de-identified healthcare organization (HCO) network, collected and analyzed the study data. Patients with fibromyalgia receiving either milnacipran or pregabalin without opioid analgesics were included in this study and separated based on their use of milnacipran or pregabalin. Risk difference, risk ratios, Kaplan-Meier analysis with log-rank tests (LRTs), hazard ratios, and proportionality were analyzed for differences among several conditions: somnolence, opioid use disorder (OUD), depressive episodes (DEs), and anxiety disorder (AD) between patients on milnacipran versus pregabalin.

Results: In total, 119,406 patients met the eligibility criteria, and propensity score matching resulted in 6,726 patients in each cohort. Milnacipran decreased the risk of somnolence and OUD (somnolence: 0.942%, 95% confidence interval (CI): 0.0445-1.44%, p<0.001; OUD: 0.779%, 95% CI: 0.061-1.5%, p<0.0333) (DEs: 2.1%, 95% CI: 0.3-3.89%, p=0.217; AD: 1.18%, 95% CI: -0.623-2.99%, p=0.119). Kaplan-Meier analysis demonstrated the benefit of milnacipran in the management of somnolence. There is no difference in the management of anxiety. There was a trending improvement in OUD and DE with milnacipran, but it did not show clear superiority (LRT somnolence, p<0.001; OUD, p=0.0584; DE, p=0.0504; anxiety, p=0.441).

Conclusion: Overall, differences observed between groups may not be clinically significant. This information may help clinicians and patients employ well-informed, shared decision-making.

## Introduction

Fibromyalgia is a complex and debilitating neck and low back pain syndrome that profoundly impacts the patient’s quality of life. It causes diffuse neuropathic or musculoskeletal pain and fatigue with disturbed sleep patterns [1,2]. The common headache is cervicogenic or at the occipital area at the insertion of the trapezius muscle. Additional symptoms may include migraines, morning stiffness, allodynia, less paresthesia, and even “fibro fog,” a form of cognitive dysfunction involving difficulties with concentration or lack of clarity of thought [3]. Roughly 2% of the US population is diagnosed with fibromyalgia, translating to roughly four million Americans [4]. The average age of onset is between 30 and 35 years, and 80% of women are impacted [5,6]. Furthermore, studies estimate that between 30% and 50%, and some up to 80%, of all patients with fibromyalgia experience depression and/or anxiety [6]. Specifically, women and younger patients with fibromyalgia are more likely to have depression, anxiety, bipolar disorder, obsessive-compulsive disorder, or personality disorders [7-12]. Other factors, including chronic illness, obesity, and traumatic events in childhood, may be associated with fibromyalgia [13-15]. Such comorbidities substantially impact patient well-being, making it imperative to address these psychological factors as part of a comprehensive treatment plan for patients with fibromyalgia.

The exact pathophysiological mechanism behind fibromyalgia remains undefined, although it is likely multifactorial in origin, including abnormal cortical processing, reductions in inhibitory pain modulatory mechanisms, and molecular changes in the pain pathway [16]. It may involve disordered nociceptive signal processing leading to sensitization [17]. Proposed neuropathic pain mechanisms include reduced noradrenergic and serotonergic signaling, disrupted dopaminergic pathways, and increased substance P activity [18,19]. Serotonin and norepinephrine can be either hyperalgesic or analgesic depending on their site of action. Peripherally, serotonin sensitizes nerve fibers, while centrally, altered 5-HT receptor activity heightens pain sensitivity. Norepinephrine suppresses nociceptors via spinal inhibition, contributing to analgesia [20]. These mechanisms support the use of serotonin norepinephrine reuptake inhibitors (SNRIs) and pregabalin, which modulate neurotransmitter release.

Current treatment options for fibromyalgia generally involve a comprehensive approach, including both pharmacological and non-pharmacological measures. One intervention, pregabalin, has been used since 2007 to provide symptomatic relief for pain [21]. However, a known side effect of pregabalin is somnolence, which can further exacerbate the fatigue that patients already experience [22]. Alternatively, the SNRI milnacipran was approved for fibromyalgia treatment in 2009 by the US Food and Drug Administration [23]. Studies showed milnacipran improved short-term outcomes of fibromyalgia-related symptoms and addressed comorbid psychiatric conditions by reducing the subjective depressive and anxiety symptoms [24,25]. For this reason, milnacipran may be superior to pregabalin for managing both pain and concomitant psychiatric conditions, with the added benefit of no exacerbation of preexisting somnolence and fatigue.

This paper explores the risk of developing four comorbidities (anxiety disorder (AD), depressive episodes (DEs), somnolence, and opioid use disorder (OUD)) in patients with fibromyalgia taking pregabalin or milnacipran. Anxiety and depressive episodes were selected due to previously mentioned documentation that indicates that these are common psychiatric disorders associated with fibromyalgia. Somnolence was selected to elucidate whether one treatment was better for managing fatigue without opioids. Lastly, despite opioids not being recommended for the treatment of fibromyalgia-related pain, studies also demonstrate that having comorbid fibromyalgia and psychiatric comorbidities increases the rate of opioid prescription for this population. The cohorts were specifically queried with no prior opioid use. Further, we assess if the time between the initial diagnosis of fibromyalgia and the subsequent diagnosis of an event of interest is substantially longer between the treatment groups. We predict that patients taking milnacipran will be less likely to have any one of the four diagnoses and will have longer latency between fibromyalgia diagnosis and psychiatric disorder onset. Overall, we hope to evaluate if milnacipran will improve psychiatric outcomes in patients with fibromyalgia and reduce the incidence of somnolence.

## Materials and methods

TriNetX is a global federated health research network with access to electronic medical records, including diagnosis and procedure billing codes, across large healthcare organizations (HCOs) in the USA. It is a large federated electronic medical record database that increases generalizability. This online research database was used to assess the presence of comorbid conditions in patients diagnosed with fibromyalgia retrospectively. All queries were limited to the US Collaborative Network in TriNetX, which included a total of 65 HCOs. Patients met the inclusion criteria if they had been diagnosed with fibromyalgia and were prescribed non-opioid analgesics to manage their pain. Those who were managed with opioid analgesics were not included. This was accomplished by using the diagnosis ICD-10 codes listed in Table 1. Patients were excluded if they did not meet the eligibility criteria for a fibromyalgia diagnosis prior to 2005. Eligible patients were then divided into two treatment groups: those who received milnacipran (N=5,694) and those who received pregabalin (N=91,286). The discrepancy in the total number can be explained by the non-reporting of one or more healthcare organizations in the TriNetX platform. Patients were excluded if their treatment history included both milnacipran and pregabalin, or if they had been prescribed a different SNRI that is also approved to treat fibromyalgia. Patients were not disqualified if they were using other pain management strategies such as non-steroidal anti-inflammatories (NSAIDs) or topical pain relievers such as diclofenac gel, as these medications can be obtained via prescription or over the counter, and it was not possible to fully evaluate whether patients were utilizing these pain management unless it was only prescribed to them. After establishing cohorts, patients were indexed using additional diagnosis ICD-10 codes for four events of interest: anxiety disorder, depressive episode, somnolence, and opioid use disorder (Table 1).

**Table 1:** Diagnostic codes used for data analysis

Diagnosis	ICD-10 code
Opioid use, abuse, and dependence disorder (opioid abuse and opioid abuse uncomplicated)	F11.22, F11.23, F11.24, F11.25, F11.28, F11.29, F11.9, F11.92, F11.93, F11.94, F11.98, F11.99, F11.150, F11.151, F11.159, F11.181, F11.182, F11.188, F11.950, F11.951, F11.959
Depressive episode	F32, F32.A, F32.0, F32.1, F32.2, F33, F33.0, F33.1, F33.2, F34.1
Somnolence	R40.0
Anxiety disorder (anxiety disorder, unspecified, and other anxiety disorders)	F06.4, F41, F41.1, F41.9

The time window for the indexed event started one day after the inclusion criteria of a fibromyalgia diagnosis were met, ensuring that patients would only be counted if their diagnosis of any psychiatric comorbidity listed in Table 1 occurred after their diagnosis of fibromyalgia. Patients were not counted if they were diagnosed with any of the psychiatric comorbidities listed in Table 1 prior to their diagnosis of fibromyalgia.

After identifying corresponding ICD-10 codes and organizing the two treatment groups, TriNetX then collected the data and calculated associated statistics. All data was collected and analyzed on December 25, 2025, at 08:36 UTC. TriNetX calculated risk reduction, risk difference, and odds ratios along with corresponding 95% confidence intervals (CIs) to assess the risk of developing psychiatric comorbidities across groups. TriNetX also performed Kaplan-Meier analysis, log-rank tests (LRTs), and hazard ratios with 95% confidence intervals and assessed proportionality to evaluate the time until patients developed a psychiatric diagnosis. P-values were also calculated by TriNetX for all forms of statistical analysis to aid in the interpretation of statistical significance relative to an alpha value of 0.05.

This retrospective study is exempt from requiring informed consent, as the patient data included in TriNetX does not contain any Protected Health Information (PHI) and is therefore not classified as human subjects research [26]. The authors assert that all procedures contributing to this work comply with the ethical standards of the relevant national and institutional committees on human experimentation and with the Helsinki Declaration of 1975, as revised in 2013. The data reviewed is a secondary analysis of existing data and does not involve any form of intervention or interaction with human subjects. Furthermore, all data obtained for the present study have been de-identified per the de-identification standard defined in Section §164.514(a) of the HIPAA Privacy Rule. The process by which the data is de-identified has been attested through a formal determination by a qualified expert as defined in Section §164.514(b)(1) of the HIPAA Privacy Rule. This formal determination by a qualified expert was refreshed in December 2020.

## Results

In total, 134,451,644 patient records were screened, and 119,406 patients met the eligibility criteria, with 112,680 patients in the pregabalin cohort and 6,726 patients in the milnacipran cohort (Table 2). The demographic information of the final cohort following propensity score matching is contained in Table 2.

**Table 2:** Propensity score matching SMD: standardized mean differences

Characteristic	Cohort	Number	% of total cohort	P-value	SMD
Total	Pregabalin	6,726	100	-	-
	Milnacipran	6,726	100		
Female	Pregabalin	6,377	94.8	0.787	0.00467
	Milnacipran	6,370	94.7		
Male	Pregabalin	349	5.19	0.938	0.00134
	Milnacipran	351	5.22		
Unknown gender	Pregabalin	0	0	0.00156	0.0546
	Milnacipran	5	0.08		
White	Pregabalin	5,482	81.5	0.147	0.02503
	Milnacipran	5,417	80.5		
Black or African American	Pregabalin	687	10.2	0.865	0.00294
	Milnacipran	694	10.3		
Asian	Pregabalin	35	0.54	0.071	0.0312
	Milnacipran	53	0.79		
American Indian or Alaskan Native	Pregabalin	26	0.40	0.369	0.0155
	Milnacipran	34	0.51		
Native Hawaiian or Other Pacific Islander	Pregabalin	9	0.15	0.53169	0.0108
	Milnacipran	13	0.19		
Other race	Pregabalin	149	2.23	0.177	0.0233
	Milnacipran	174	2.59		
Unknown race	Pregabalin	338	5.04	0.937	0.00136
	Milnacipran	341	5.07		
Hispanic or Latino	Pregabalin	377	5.61	0.940	0.00129
	Milnacipran	375	5.58		
Not Hispanic or Latino	Pregabalin	5,017	74.6	0.937	0.001367
	Milnacipran	5,021	74.7		
Unknown ethnicity	Pregabalin	1,332	19.8	0.965	0.00136
	Milnacipran	1,330	19.8		

The risk of a psychiatric outcome for patients on pregabalin and milnacipran was 2.63% and 1.68% for somnolence, 4.87% and 4.09% for opioid use disorder (OUD), 20.6% and 18.5% for depressive episodes, and 22.2% and 21.1% for anxiety, respectively (Table 3).

**Table 3:** Risk differences CI: confidence interval

	Risk (%)	Risk difference (%)	95% CI	z	p
Somnolence					
Pregabalin	2.63	-0.942	(-1.44, -0.0445)	-3.72	<0.001
Milnacipran	1.68				
Opioid use disorder					
Pregabalin	4.87	-0.779	(-1.50, -0.061)	-2.13	0.0333
Milnacipran	4.09				
Depressive episode					
Pregabalin	20.6	-2.10	(-3.89, -0.304)	-2.30	0.217
Milnacipran	18.5				
Anxiety					
Pregabalin	22.2	-1.18	(-2.99, -0.623)	-1.28	0.199
Milnacipran	21.1				

**Figure 1:**
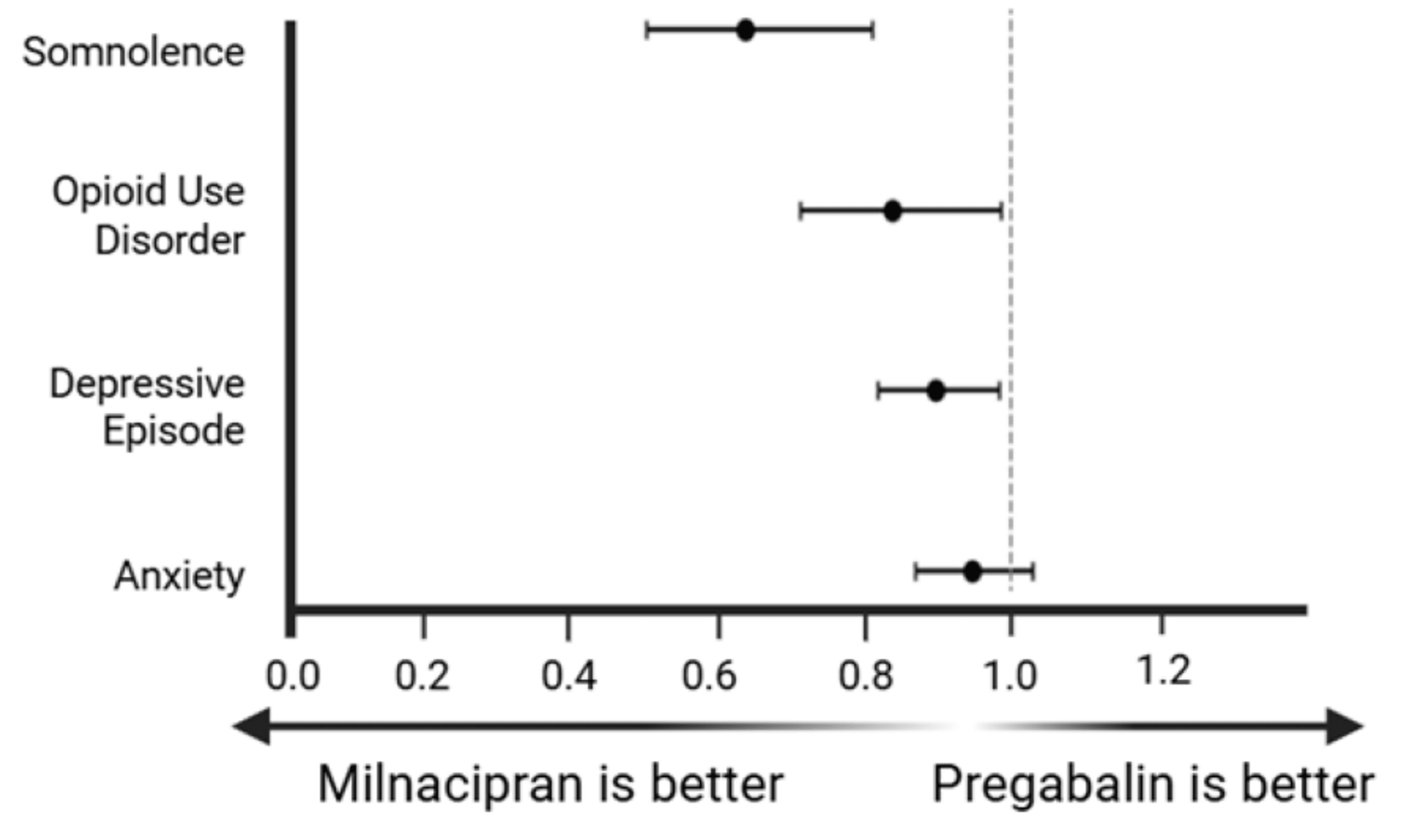
Risk ratios (95% CI) of select psychological comorbidities in patients with fibromyalgia treated with either milnacipran or pregabalin (somnolence: 0.641 (0.506-0.812), opioid use disorder: 0.840 (0.715-0.986), depressive episode: 0.898 (0.820-0.984), anxiety: 0.947 (0.871-1.03)) CI: confidence interval

Risk analysis showed a statistically significant decrease in risk of somnolence (risk difference: 0.942% (95% CI: 0.0445-1.44%), p<0.001) and OUD (risk difference: 0.779% (95% CI: 0.061-1.5%), p=0.0333), but not in depressive episodes (risk difference: 2.1% (95% CI: 0.304-3.89%), p=0.217) or anxiety disorders (risk difference: 1.18% (95% CI: 0.623-2.99), p=0.199). Risk ratios (95% CI) when comparing milnacipran to pregabalin groups showed a reduction of somnolence (0.641 (0.506-0.812)), OUD (0.840 (0.715-0.986)), and depressive episodes (0.898 (0.820-0.984); Figure 1). No significant difference in risk ratio was found for anxiety (0.947 (0.871-1.03); Figure 1).

**Figure 2:**
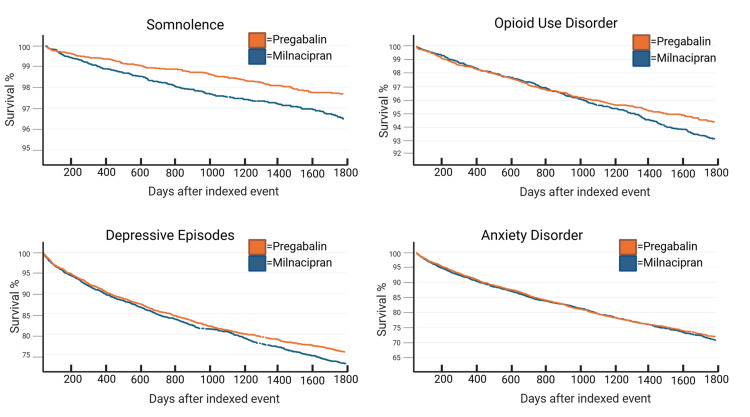
Kaplan-Meier analysis Somnolence survival at the end of the time window: pregabalin, 96.4% (95.9-97.0); milnacipran, 97.6% (97.2-98.1) Opioid use disorder survival at the end of the time window: pregabalin, 93.2% (92.4-93.9); milnacipran, 94.4% (93.7-95.1) Depressive episode survival at the end of the time window: pregabalin, 72.8% (71.1-74.6); milnacipran, 75.5% (74.1-77.3) Anxiety disorder survival at the end of the time window: pregabalin, 70.8% (69.3-72.7); milnacipran, 72.0% (70.4-73.7)

Across our psychiatric events of interest, Kaplan-Meier analysis demonstrated variation in survival at 1,800 days. The largest difference was seen in the diagnosis of depression, with roughly 27% of patients in the pregabalin group diagnosed with depression at the end of the time window compared to roughly 24% of patients in the milnacipran group (Figure 2).

However, log-rank tests (LRTs) and hazard ratios (HRs) indicate that this difference is not statistically significant (LRT, p=0.0504; HR: 0.903, 95% CI: 0.815-1; Table 4). Kaplan-Meier curves indicated a 1.3% difference in the final number of patients diagnosed with somnolence (Figure 2). This difference was statistically significant, and survival curves remained proportional over time (LRT, p<0.001; HR: 0.646, 95% CI: 0.509-0.820; proportionality, p=0.450; Table 4). Patients treated with pregabalin had a 1.2% increase in the number of patients diagnosed with OUD (Figure 2). While hazard ratios indicate that this difference was not statistically significant, proportionality tests indicate that the difference in hazard ratios was not equal throughout the duration of the study (HR: 0.853, 95% CI: 0.724-1.01; proportionality, p=0.0129; Table 4). Lastly, there was minimal difference in the number of patients diagnosed with anxiety by the end time point, with the pregabalin group having 1% more patients than the milnacipran group (Figure 2). Log-rank tests, hazard ratios, and proportionality tests all show that this difference was insignificant (LTR, p=0.441; HR: 0.964, 95% CI: 0.877-1.06; proportionality, p=0.379; Table 4).

**Table 4:** Analysis of time until patients with fibromyalgia receive a psychiatric diagnosis: comparing log-rank test and hazard ratios for patients with fibromyalgia treated with milnacipran or pregabalin across different psychiatric comorbidities: somnolence, opioid use disorder, depressive episodes, and anxiety disorder CI: confidence interval

	Log-rank test			Hazard ratio		Proportionality		
Event of interest	χ^2^	df	p	Hazard ratio	95% CI	χ^2^	df	p
Somnolence	13.1	1	<0.001	0.646	(0.509, 0.820)	0.570	1	0.450
Opioid use disorder	3.58	1	0.0584	0.853	(0.724, 1.01)	6.18	1	0.0129
Depressive episode	3.83	1	0.0504	0.903	(0.815, 1.00)	1.55	1	0.213
Anxiety disorder	0.595	1	0.441	0.964	(0.877, 1.06)	0.775	1	

## Discussion

The lack of adequate pharmacological interventions for the management of fibromyalgia is often a hard pill to swallow for patients. While further investigation of treatments that address fibromyalgia’s pathophysiology is necessary, studies like ours are needed to explore optimal treatments to manage symptoms in the interim. Milnacipran may be a reasonable alternative for patients with fibromyalgia and comorbid psychiatric conditions, but it needs further research for clinical decision-making. Risk analysis comparing the pregabalin and milnacipran treatment groups showed a 1% reduction in somnolence diagnosis within the milnacipran group (p<0.001; Table 3). Our Kaplan-Meier analysis showed that fewer patients in the milnacipran group had been diagnosed with somnolence and that this difference was significant (LTR, p<0.001, HR: 0.646, 95% CI: 0.509-0.820; proportionality, p=0.450; Table 4). However, we expected there to be a greater difference in somnolence diagnosis between the two groups. A possible explanation is that clinicians did not diagnose patients with somnolence, as it is both a common symptom of fibromyalgia and a side effect of pregabalin. This would limit our ability to assess meaningful differences across both groups and cause us to underestimate the number of patients who experience somnolence, ultimately masking the potential benefits of milnacipran.

Milnacipran marginally reduced the risk of OUD. Risk analysis indicated that this finding was significant, but HR’s indicated that over time, this difference was insignificant and the degree of any potential benefit was inconsistent (p=0.0333, Table 3; OUD: RR=0.840, 95% CI: 0.715-0.986, Figure 1; OUD: pregabalin, 93.2%, Figure 2; milnacipran: 94.4%; LRT, p=0.0584, HR: 0.853, 95% CI: 0.724-1.01; proportionality, p=0.0129, Table 4). For these reasons, the clinical significance of these findings is debatable. An important consideration is that OUD carries risks for morbidity and mortality, so any amount of risk reduction may be beneficial for patients [27]. Further, the risk of opioid use disorder may be higher in the general fibromyalgia population than in our study cohort, as people previously prescribed opioids were excluded from analysis. This may have eliminated patients at increased risk of developing OUD, leading to an underestimation of the true risk reduction and impacting our ability to generalize findings.

There were no significant differences found between treatment groups for anxiety and depression with the exception of a marginal reduced risk ratio of depressive episodes in patients taking milnacipran (anxiety risk difference: 1.18%, p=0.199, depressive episode risk difference: -2.1%, p=0.217, Table 3; anxiety risk ratio: 0.947, 95% CI: 0.871-1.03, depressive episode risk ratio: 0.898, 95% CI: 0.820-0.984, Figure 1; anxiety: LRT, p=0.441, HR: 0.964, 95% CI: 0.877-1.06; depression: LRT, p=0.0504, HR: 0.903, 95% CI: 0.815-1, Table 4). Taken together, there is no statistical evidence of milnacipran managing comorbid anxiety or depression despite SNRIs being used to treat these psychiatric conditions [28]. This may indicate that the benefit of SNRIs in fibromyalgia treatment is limited to modulating neuropathic pain as opposed to psychiatric comorbidity. Unfortunately for patients, this may mean that the ultimate treatment for their pain and psychiatric symptoms must come from multiple concurrent treatments instead of being managed by a single SNRI. This is important, as milnacipran in combination with other serotonin-based medications can lead to serotonin syndrome and can cause other side effects such as worsening depression and suicidal ideation [29]. These are all factors clinicians should consider when selecting the best treatment option for managing anxiety and depression in patients with fibromyalgia.

While this study demonstrates that milnacipran may be equivalent to pregabalin in terms of managing the four diagnoses of interest, milnacipran does not impart significant benefit either. Select patients may benefit from a reduction in somnolence and OUD, but the differences identified in this study are small and are likely not significant enough to influence clinical decision-making. Further, per the findings demonstrated in this study, it is unlikely that patients would benefit from managing both their fibromyalgia-related pain and concomitant psychiatric conditions, such as depressive episodes or anxiety. This suggests the need for additional therapies in patients with coexisting fibromyalgia and anxiety or depression. This study has inherent limitations, as multiple factors impact the accuracy of ICD-10 coding analysis. First, different providers may select differing billing codes for similar diagnoses, which would alter our cohorts and analyses. Providers may also not code for acute psychiatric episodes for patients with long-term conditions. Second, our analysis cannot account for medication compliance. It is possible that patients in either group were not taking their medications as prescribed, leading to suboptimal treatments. Reasons for noncompliance include barriers to accessing their medications, such as transportation issues, financial strain, and insurance status. Furthermore, our investigation did not explore other side effects of these drugs, which may make one more tolerable than the other and have an impact on medication compliance. We have not assessed theoretical confounding variables, such as race, sex, and socioeconomic status, among others. It is possible that the intersectionality of these personal identifiers, combined with having a chronic disease, would lead to a difference in patient outcomes. This could be reflected by differences in access to healthcare centers, medications, transportation, and other parameters previously described.

Future research could investigate the effect of non-pharmacological therapies, such as aquatic therapy, aerobic conditioning, and electrical nerve stimulation, on resolving fibromyalgia-related pain and any potential impact on either the development of new psychiatric comorbidities or the resolution of previously diagnosed psychiatric conditions that may prove to be beneficial. Furthermore, assessing variations within demographic categories, including race, sex, and gender, may prove to be valuable. Lastly, a study assessing the incidence and prevalence of opioid use disorder in patients with fibromyalgia receiving opioid-based treatments might give more insight, as this patient population might have an increased risk for developing substance use disorder. Continued research is crucial to gain a better understanding of fibromyalgia and find improved ways to provide symptomatic relief to patients with this debilitating disease.

## Conclusions

As researchers continue to elucidate the etiology of fibromyalgia, better medications may arise to better assist these patients. Until then, the present study dealt with the psychiatric comorbidities of fibromyalgia and various methods to treat it, but not its complex and multifaceted nature. Our data largely demonstrates equivalence between milnacipran and pregabalin. While more research is needed to find permanent and sustainable clinical solutions and outcomes, care for these patients should continue to be individualized and tailored to each patient’s unique needs.
